# Oxidative stress and mitochondrial dysfunction of granulosa cells in polycystic ovarian syndrome

**DOI:** 10.3389/fmed.2023.1193749

**Published:** 2023-06-28

**Authors:** Yiyin Gao, Yinggang Zou, Guijie Wu, Lianwen Zheng

**Affiliations:** Reproductive Medical Center, Department of Obstetrics and Gynecology, The Second Hospital of Jilin University, Changchun, China

**Keywords:** polycystic ovarian syndrome, oxidative stress, granulosa cells, mitochondrial function, reactive oxygen species, infertility

## Abstract

Polycystic ovarian syndrome (PCOS) is one of the leading causes of anovulatory infertility in women, affecting 5%–15% of women of reproductive age worldwide. The clinical manifestations of patients include ovulation disorders, amenorrhea, hirsutism, and obesity. Life-threatening diseases, such as endometrial cancer, type 2 diabetes, hyperlipidaemia, hypertension, and cardiovascular disease, can be distant complications of PCOS. PCOS has diverse etiologies and oxidative stress (OS) plays an important role. Mitochondria, as the core organelles of energy production, are the main source of reactive oxygen species (ROS). The process of follicular growth and development is extremely complex, and the granulosa cells (GCs) are inextricably linked to follicular development. The abnormal function of GCs may directly affect follicular development and alter many symptoms of PCOS. Significantly higher levels of OS markers and abnormal mitochondrial function in GCs have been found in patients with PCOS compared to healthy subjects, suggesting that increased OS is associated with PCOS progression. Therefore, the aim of this review was to summarize and discuss the findings suggesting that OS and mitochondrial dysfunction in GCs impair ovarian function and induce PCOS.

## 1. Introduction

Polycystic ovarian syndrome (PCOS) is a common reproductive endocrine disorder, in which patients often present with ovulatory disturbances, amenorrhoea, and androgen excess disorders (1). PCOS in adults is diagnosed when at least two of the following three criteria are met: impaired ovarian function, clinical and/or biochemical hyperandrogens, and polycystic ovaries (2). Despite a long history of PCOS research, its pathogenesis remains unclear. It is widely believed that dysregulation of androgen and gonadotropin secretion, oxidative stress (OS), abnormal mitochondrial function and glucose metabolism disorders lead to impaired energy and metabolic regulation, and insulin resistance (IR) and hyperandrogenism are core factors in PCOS etiology (3).

Molecular oxygen (O_2_) is essential for all aerobic organisms. There are two unpaired electrons in different orbitals in the outer shell of O_2_, which facilitates radical formation (4). Superoxide (O_2_^−^) is the reduced product of O_2_ and the precursor of other reactive oxygen species (ROS). Superoxide anions (O_2_^−^), hydroxyl radicals (OH^−^), peroxyls (ROO), alkoxyls (RO), and hydroperoxyls (HO_2_) are the most biologically important ROS (5). Appropriate ROS levels play an important role in the oxidative modification of proteins, such as receptors, ion channels, and transcription factors (6, 7). However, excessive ROS levels cause an imbalance in the redox system and lead to OS, which, in turn, has cytotoxic effects by initiating the peroxidation of membrane phospholipids and altering nucleic acids, lipids, and proteins. These processes lead to altered cell physiology, including apoptosis, increased membrane permeability, reduced enzyme activity, DNA damage, mitochondrial alterations, and ATP depletion (8, 9).

The process of follicular growth and development is complex and delicate, and granulosa cells (GCs) are inextricably linked to this process (10). Abnormalities in GC function may directly affect follicular development and alter several features of PCOS (11). OS may alter the function of GCs and oocytes and affect the follicular fluid (FF) microenvironment, as well as ovarian bioactivities, such as hormonal signaling, oocyte maturation, and ovarian steroidogenesis, and eventually have a negative impact on reproductive outcomes (12).

OS is considered a potential trigger for the development of PCOS, as numerous studies have shown that markers of oxidative damage are significantly elevated in the serum of patients with PCOS compared to those in normal subjects (13, 14). There are several mechanisms underlying OS in the development of PCOS. In this review, we focused on the OS of GCs in the pathogenesis of PCOS by reviewing *in vitro* and *in vivo* studies. Understanding the role of OS in PCOS may provide future directions for the development of treatment strategies.

## 2. GCs and their role in PCOS

Ovarian GCs are round or oval in shape and closely distributed around the oocyte, which comprises the cellular components of the follicle together with the oocyte; corpus luteum cells constitute the cellular components of the follicle (15, 16). As the largest cell population in the follicle, the proliferation and differentiation status of GCs directly affect the quality of oocytes, which consequently affects the functional activities of the ovary, such as the initiation and development of follicles, ovulation, and corpus luteum formation (17). Mitochondrial function and developmental potential of oocytes can be enhanced by surrounding GCs via mitophagy (18). In the oocyte-GC complex, low molecular weight signals or nutrient molecules are transferred between cells as a means of transporting nutrients to the oocyte and sharing metabolic intermediates and products. The transmission capacity increases as the ratio of oocyte volume to surface area increases (10). As the oocyte grows, the zona pellucida gradually separates GCs from the oocyte, and the GCs contact the oocyte by producing filamentous pseudopods that cross the zona pellucida (19). Thus, oocytes acquire glycolytic and cholesterol metabolites from GCs, which cannot be synthesized independently (20). The growth and development of oocytes depend on the production of such metabolites by GCs and their transfer across the zona pellucida to the oocyte, which is known as metabolic coupling. The greater the number of GCs that are metabolically coupled to the oocyte, the faster the oocyte grows (21). A distinctive sign of follicular development is the rapid growth and proliferation of GCs, whereas follicular atresia is mainly caused by the apoptosis of GCs. In addition, GCs work together with follicular membrane cells to complete the synthesis of ovarian hormones and maintain a microenvironment conducive to oocyte growth and maturation (22).

GCs play a supportive role in physiological folliculogenesis and greatly influence pathological folliculogenesis in PCOS, premature ovarian failure (POF), and some malignant diseases, such as ovarian granulosa cell tumors (GCT) (11). Abnormalities in GC function may directly affect follicular development and alter several PCOS features. In PCOS patients, it is difficult for multiple small antral follicles to develop into larger dominant follicles. In addition, the function of GCs differs in patients with PCOS. A significant increase in follicle number and proliferation of GCs was observed in a PCOS mouse model, as well as in the ovaries of patients with PCOS (23, 24). Therefore, abnormal proliferation of ovarian GCs is likely to play a role in the pathogenesis of PCOS. Furthermore, previous studies have found that increased levels of apoptosis in GCs are one of the key factors contributing to follicular dysplasia and impaired oocyte maturation. Peng found that the level of apoptosis in GCs was significantly elevated in patients with PCOS, and that knocking down the expression of PATL2, a gene associated with oocyte development, using siRNA significantly prevented apoptosis of GCs (25). In addition, the anti-Müllerian hormone (AMH) secreted by GCs inhibits sinus follicle development, and decreased AMH levels are a prerequisite for ovulation. In contrast, androgens in the follicular fluid of patients with PCOS can block the decrease in AMH levels either directly or through the AMH-Smad signaling pathway. Moreover, GCs have a reduced ability to respond to persistently elevated AMH levels, resulting in follicular development arrest (26).

Since patients with PCOS may be affected by obesity and hyperinsulinemia, they often exhibit a chronic inflammatory state. Evidence suggests that OS and chronic inflammation caused by mitochondrial damage in GCs are associated with the pathogenesis of PCOS. A study investigating the metabolomics of FF in PCOS showed that mitochondrial dysfunction of GCs, redox potential imbalance, and increased cellular OS levels in PCOS patients could explain, to some extent, the metabolic disorders observed in PCOS (27).

## 3. Markers of OS in PCOS

OS refers to the excessive production of ROS *in vivo* or impaired production of antioxidants, resulting in an imbalance between oxidants and antioxidants, which may lead to DNA damage and apoptosis (28). ROS represent a class of molecules that originate from the metabolism of oxygen in aerobic organisms and include the one-electron reduction product of oxygen, superoxide anion (O_2_^−^), two-electron reduction product, hydrogen peroxide (H_2_O_2_), three-electron reduction product, hydroxyl radical (OH), and nitric oxide (NO) (28). ROS function as intracellular secondary messengers by regulating signal transduction pathways, gene expression, post-translational modification of proteins, and participation in cell proliferation, differentiation, and apoptosis (29). Highly complex antioxidant enzymes and nonenzymatic systems regulate ROS scavenging. Various antioxidant enzymes, such as superoxide dismutase (SOD), catalase (CAT), glutathione peroxidase (GPx), glutathione reductase (GR), glucose 6-phosphate dehydrogenase and isocitrate dehydrogenase can attenuate OS and tissue damage in cells by scavenging ROS (30). However, the main mechanism of resistance to excessive ROS damage is the nonenzymatic antioxidant system. It contains antioxidants, such as vitamins and polyphenols, and directly neutralizes ROS in the body (31). Trace elements, such as Cu, Zn, and Mn, are cofactors for antioxidant enzymes and are essential components of the enzymatic antioxidant system (30).

The detection of OS and antioxidant biomarkers has been suggested as a useful tool for assessing the risk of oxidative damage in various diseases (32). Table 1 shows the results of several OS markers detected in patients with PCOS in different studies.

**Table 1:** Oxidative stress markers in patients with PCOS.

Biomarkers	Origin	Changes in PCOS patients
MDA	Serum; Erythrocyte	↑ (33, 34)
NO	Serum	↑ (35) or (36, 37)
AGEs	Serum	↑ (38)
XO	Serum	↑ (36, 39)
TOS	Serum, FF	↑ (40)

MDA, malondialdehyde; NO, nitric oxide; AGEs, advanced glycation end products; XO, xanthine oxidase; TOS, total oxidant status.

Malondialdehyde (MDA) is a stable biomarker of lipid peroxidation, as reported in several studies of patients with PCOS. Circulating MDA concentrations increased by 47% in patients with PCOS compared to controls (41). In addition, Palacio et al. confirmed that MDA levels in erythrocytes were significantly higher in patients with PCOS than in BMI-and age-matched control populations (42). Another intracellular signaling molecule, NO, is involved in many biological processes and can be toxic in high concentrations. Although a meta-analysis revealed no significant difference in NO levels in patients with PCOS compared to controls (41), another study by Nacul et al. reported a significant negative correlation between NO levels and IR-related indices, including fasting insulin levels and homeostatic model assessments in patients with PCOS (35). AGEs are a class of stable end products formed by the non-enzymatic reaction of free amino groups of molecules, such as proteins, amino acids, and lipids with aldehyde groups of reducing sugars. AGEs have been reported as markers of OS in PCOS, and there is growing evidence that AGEs may alter steroid biosynthesis in PCOS by affecting enzyme function, inducing inflammatory changes and IR, and ultimately affecting androgen synthesis and follicular development (42). Tantalaki et al. investigated the effect of dietary intake of AGEs on hormone secretion. The results indicated that PCOS patients with a diet high in AGEs had higher levels of serum AGEs along with elevated levels of testosterone, free androgen index, and androstenedione, confirming the association between AGEs and excessive androgen levels (43). XO is an enzyme that participates in O_2_^−^ generation, which is also involved in the metabolism of purines and catalyzes the production of ROS (36). Isık et al. found that serum C-reactive protein (CRP), platelet and platelet-specific volume (PCT) values, and XO activity were significantly higher in patients with PCOS, while SOD activity was decreased. XO activity was positively correlated with fasting plasma insulin and IR index and negatively correlated with quantitative insulin sensitivity check index levels. Therefore, XO may be used as a sensitive marker to assess the OS of patients with PCOS (44).

Generally, several cellular mechanisms defend against free radicals in the organelles, cell membranes, and extracellular environment. One mechanism involves enzymes, such as CAT, GPx, and SOD, which prevent the production or rapidly inactivate free radicals. Another mechanism interrupts the propagation of the lipid peroxidation chain by reacting with intermediate free radicals, which work as scavenging molecules, including albumin, bilirubin, ascorbic acid, fat-soluble vitamin E, and coenzyme Q10. Finally, the process of scavenging molecules damaged by oxidative attack is used to re-establish normal structure. Table 2 indicates several antioxidant markers in PCOS, but most of the findings are conflicting and further studies are required to elucidate the mechanisms underlying the changes in antioxidant levels in PCOS patients.

**Table 2:** Anti-oxidative stress markers in patients with PCOS.

Biomarkers	Origin	Changes in PCOS patients
SOD	Serum; Erythrocyte; FF	↑ (39) or↓ (39, 45, 46)
GPx	Serum	↓ (47) or (36)
CAT	Serum	↓ (45)
TAC	FF, Serum	↑ (45) or ↓ (44, 48) or (37)
GSH	Serum	↓ (45)
NAD+	GCs	↓ (49)
Vitamin E	Serum	↓ (50)
Vitamin C	Serum	↓ (50)

SOD, superoxide dismutase; GPx, glutathione peroxidase; TAC, total antioxidant capacity; GSH, glutathione; NAD+, nicotinamide adenine dinucleotide.

SOD catalyzes the production of H_2_O_2_ from O_2_^−^, which is further converted to H_2_O by GPx, thereby eliminating ROS and performing its antioxidant function. Serum SOD activity in patients with PCOS has been reported to differ, with Sabuncu et al. claiming that serum SOD was significantly higher in patients with PCOS than in controls, whereas Zhang et al. reached the opposite conclusion (51, 52). Seleem et al. reported lower SOD activity in both the serum and FF of patients with PCOS and concluded that serum SOD activity may be a clinical parameter for determining systemic OS in PCOS (44). Further studies are required to explore the antioxidant defense mechanisms of SOD in PCOS. However, neither study showed that GPx activity in patients with PCOS differed from that in controls (36, 52), although Savic-Radojevic et al. reported significantly lower GPx activity in patients with PCOS than in controls (53).

Total antioxidant capacity (TAC) refers to the ability of extracellular nonenzymatic antioxidants to scavenge free radicals, which can block the propagation chain of lipid peroxidation and prevent the expansion of free radical generation and subsequent biochemical damage (54). TAC is regarded as a better indicator of the total antioxidant status of a biological sample because it reflects the combined effects of various antioxidants (55). Several studies have reported TAC levels in patients with PCOS. A meta-analysis showed no significant difference in TAC between patients with PCOS and controls (53); however, Fenkci et al. showed significantly lower TAC levels in patients with PCOS (56). Verit et al. reported significantly elevated TAC levels in patients with PCOS compared to age-and BMI-matched controls (57). Although the complete mechanism underlying this elevation is unknown, they speculated that elevated TAC levels may be a responsive change in the body in answer to increased OS levels (57). As the most important non-enzymatic antioxidant, GSH is essential for regulating protein disulfide bonds and scavenging oxidants. Dinger et al. suggested that GSH depletion may be due to increased ROS production in patients with PCOS (58). NAD+ is involved in the regulation of the energy tricarboxylic acid cycle, oxidative phosphorylation, and intracellular glycolytic reactions, and it is often considered as a central component of redox reactions with its reduced form, NADH (59). It has been shown that NAD+ levels in GCs of women with PCOS are lower than in controls, accompanied by elevated levels of inflammatory factors and abnormal mitochondrial dynamics (49). In addition, it has also been shown that concentrations of vitamin C and vitamin E, which have antioxidant capacity, are lower in PCOS patients than in controls (50).

## 4. Etiology related to OS in PCOS

Increased levels of ROS in ovarian GCs of women with PCOS may impair the function of mitochondrial oxidative metabolism, leading to abnormal morphology of GCs, resulting in inadequate energy supply and increased OS, while greatly inducing apoptosis of GCs. Elevated levels of ROS in FF are associated with poor oocyte and embryo quality (60). In turn, altered oocyte quality or capacity is thought to be a major influence on infertility or poor pregnancy outcomes in women with PCOS (61). Although more oocytes can be collected from women with PCOS, the rates of MII oocyte, 2PN fertilization, oogenesis, day 3 quality embryo and clinical pregnancy are lower than controls (12, 62).

OS plays an important role in the development of PCOS by influencing cellular functions in several ways. Various protein kinases are reportedly activated with increased OS damage, which in turn induces serine/threonine phosphorylation of insulin receptor substrates, leading to their degradation and reduced insulin sensitivity, thereby promoting the development of IR (63). However, with elevated ROS levels, large amounts of Ca^2+^ are released from the endoplasmic reticulum, leading to the dysregulation of intracellular calcium homeostasis and cellular necrosis. Calcium dysregulation eventually causes follicular arrest in patients with PCOS, which in turn leads to reproductive disorders, such as amenorrhoea and anovulation (64). The direct oxidation of proteins and free amino acid side chains leads to the formation of carbonyl products, resulting in protein denaturation and inactivation (65). Serum levels of advanced oxidation protein products (AOPPs) are significantly higher in PCOS patients than in healthy women, and therefore AOPPs may serve as a new marker of oxidant-mediated protein damage (66). Lipid peroxidation that occurs in the side chains of polyunsaturated fatty acids in the cell membrane is often a chain reaction, and the free radicals formed by the binding of fatty acid side chains to oxygen can, in turn, bind to other fatty acids to obtain hydrogen ions (67). Lipid peroxidation markers, such as thiobarbituric acid-reactive substances, oxidized low-density lipoproteins, and MDA, were detected at significantly higher levels in patients with PCOS than in normal controls. Serum lipid concentrations increase simultaneously with peroxidation (68).

## 5. Mitochondrial functions of GCs in PCOS

Mitochondria are important for energy production and insulin secretion and are essential regulators of apoptosis because they provide energy for normal cellular functions (69, 70). Mitochondrial abnormalities usually manifest as diseases of the entire organism and lead to varying degrees of metabolic disorders (71). The results of a transcriptomic study showed altered expression of several genes involved in oxidative phosphorylation, mitochondrial function, and the sirtuin signaling pathway in the GCs of patients (72). However, little is known about the roles of mitochondrial regulatory factors in GCs.

As the powerhouse of the cell, mitochondria replenish ATP through oxidative phosphorylation (OxPhos) to maintain cellular energy homeostasis (73). Additionally, mitochondria produce ROS through the electron transport chain (ETC) complex, and excessive ROS production induces OS, leading to mitochondrial dysfunction and, consequently, an imbalance between the oxidant and antioxidant systems. The main ROS in the mitochondria is O^2−^, which is generated by electron leakage from the ETC and can react with O_2_ (74). Mitochondria contain their own genetic material, mitochondrial DNA (mtDNA), which encodes seven genes in ETC complex I (75). Mutations in mtDNA can lead to mitochondrial dysfunction and induce IR by affecting OS homeostasis, energy deficiency, or hormone metabolism. Mutations in mtDNA mostly occur in mt-tRNA genes, which alter RNA processing by destabilizing tRNA tertiary structures, leading to defects in nucleotide modifications. Defects in mitochondrial protein synthesis reduce ATP production in GCs and pancreatic cells, resulting in the clinical phenotype of PCOS. The effects of mtDNA copy number and GC variation on the regulatory mechanisms of GC function have been the focus of scientific research. Currently, the role of mtDNA copy number in PCOS is contradictory, as different groups have reported positive, negative, or no associations between mtDNA copy number and PCOS (76–78). Au et al. demonstrated that the occurrence of mitoDNA 4,977 bp deletion in GCs is associated with apoptosis (79). Overall, 33 mtDNA mutations were identified in patients with PCOS, and the majority of these mutations were found in the D-loop region (80).

**Figure 1:**
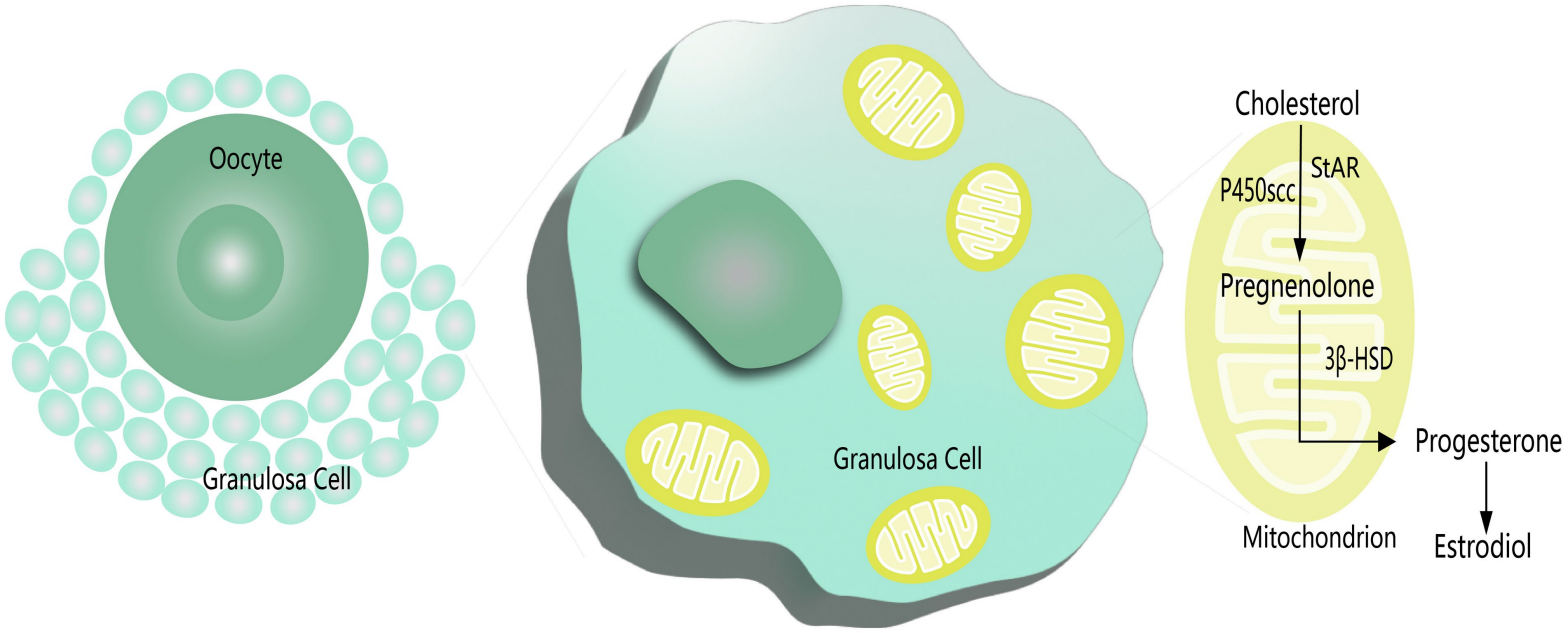
Steroidogenesis in mitochondrion of GCs.

In addition, mitochondrial swelling has been found in degenerated GCs and is associated with apoptosis and follicular atresia (81). Karuputhula et al. found reduced GC counts in patients with endometriosis and PCOS compared to controls, accompanied by decreased cell viability and mitochondrial abnormalities (82). Similar findings have been reported for mitochondrial dysfunction and mutations in GCs from patients with endometriosis or PCOS (83, 84). One of the representative functions of GCs is steroid hormone secretion. Mitochondria are the central organelles for steroidogenesis, with the initiating and rate-limiting steps of biosynthesis occurring in the mitochondria (Figure 1) (85). Studies have shown that estradiol (E_2_) levels are significantly reduced in the serum and FF of patients with endometriosis, which is associated with reduced mitochondrial quality and mitochondrial membrane potential (MMP) in GCs accompanied by elevated apoptosis in GCs (86). Gong et al. also observed decreased MMP and elevated apoptosis in GCs with higher TOS in serum and FF in patients with PCOS, while treatment with growth factor may alleviate OS and improve GCs function (40).

GCs treated with methamphetamine (MA), a central nervous system stimulant, showed significant swelling and degeneration of the mitochondria with significant enlargement of the mitochondrial cristae septum accompanied by a decline in ovarian function (87). Similarly, most GCs exposed to ozone have swollen mitochondria and display vacuolisation and degeneration of the cristae and matrix. 4-Hydroxynonenal (4-HNE), produced during lipid oxidation, modifies mitochondrial proteins, leading to mitochondrial dysfunction. 4-HNE immunostaining was significantly increased in the GCs of the ozone-exposed group, suggesting a significant impairment of mitochondrial function (88). Decreased mitochondrial function directly leads to decreased GC function, which in turn leads to decreased oocyte quality and ovarian reserve function and has a negative impact on reproductive outcomes.

## 6. Glucose metabolism of GCs in PCOS

Another downstream mechanism that plays an important role in PCOS is glucose metabolism, which also participates in the bidirectional signaling between GCs and oocytes. Since oocytes have low glycolytic activity, they preferentially utilize pyruvate and lactate produced by GCs via the mitochondrial OxPhos pathway to meet their energy requirements (89). In contrast, impaired glycolytic metabolism of GCs leads to insufficient energy substrates for oocytes and GCs, resulting in excessive ROS production due to dysfunctional mitochondrial OxPhos and ultimately impairing oocyte quality.

A recent multi-omics analysis of GCs in PCOS identified abnormalities in metabolism-related signals, such as glycolysis/glyoxylate production (90). Under hypoxic conditions, cells use the glycolytic pathway to produce ATP instead of the oxygen-independent metabolic pathway, which contributes to cell proliferation of GCs and oocyte development during hypoxia (91, 92). As a regulator of the cellular response to hypoxia, the expression of hypoxia-inducible factor 1a (HIF-a) was increased under hypoxic conditions that induces a metabolic switch from OxPhos to glycolysis (93). The reduced mitochondrial activity of GCs in PCOS patients may inhibit the signaling of HIF-1a, delaying the switch in energy metabolism during follicle growth and affecting normal follicle development (94).

Researchers have observed a significant decrease in glycolytic rate and reduced expression of key glycolytic enzymes in GCs of PCOS patients including hexokinase, muscular phosphofructokinase, platelet derived phosphofructokinase, and muscular pyruvate kinase (95). Besides, oocytes from women with PCOS exhibit increased mitochondrial ROS and decreased MMP with downregulation of glucose-6-phosphate dehydrogenase (G6PD) (12). Mazloomi et al. postulated that increased ROS levels in GCs impair glycolysis, reduce energy levels, and ultimately disrupt GCs’ function since ROS levels were inversely correlated with the expression of key glycolytic enzymes in PCOS. Zhang et al. found that these alterations were associated with a deficiency of Sirtuin 3 (SITR3) in GCs, which resides primarily in mitochondria and regulates mitochondrial proteins via deacetylation (96). SIRT3 in GCs is involved in processes such as metabolic homeostasis and OS by targeting mitochondrial enzymes, such as glutamate dehydrogenase (97). In patients with PCOS, the deficiency of SIRT3 in GCs may lead to elevated mitochondrial ROS and altered cellular insulin signal pathways by altering the acetylation status of NDUFS1. The incapacity of mitochondria in GCs to maintain ATP synthesis promotes OS and impairs glucose metabolism, and eventually negatively influence the maturation of oocyte (12).

## 7. Treatment strategy of PCOS

Given the role of mitochondrial dysfunction in the development of PCOS, treatments targeting the mitochondria to improve PCOS symptoms have become a research hotspot. Vitamin D can directly act on mitochondria, which improves cell biosynthesis and anti-apoptosis levels, and upregulates antioxidant expression, thereby reducing ROS levels. Moreover, vitamin D stimulates the expression of mitochondrial transcription factor A, increases mtDNA copy number, and improves the integrity of the mitochondrial membrane (98). In DHEA-induced PCOS rat models, vitamin D supplementation improved ovarian and uterine morphology, and reduced body weight and obesity (99). Additionally, vitamin D regulates steroid production and reduces progesterone (P) and E_2_ production by reducing the expression of steroidogenic enzymes. The application of the mitochondria-targeted antioxidant MitoQ10 can improve IR status, reduce the expression levels of apoptosis-related proteins, and improve damaged mitochondrial function in PCOS rats (100).

The mitochondria-targeted antioxidant melatonin enhances SIRT1 expression, thus regulating PINK1/Parkin-mediated mitochondrial autophagy. The application of melatonin in PCOS patients significantly improves mitochondrial damage in GCs (101). Growth hormone can alleviate OS-induced apoptosis in GCs of patients with PCOS by activating the PI3K/Akt signaling pathway. The acute-phase protein PTX3 is locally expressed at the site of inflammation and can serve as a sensitive marker of endothelial dysfunction in patients (102). PTX3 is synthesized by GCs and plays an important role in normal oocyte development and fertilization. In contrast, PTX3 levels in the follicular fluid of PCOS patients are positively correlated with serum androgen concentrations, suggesting that the chronic inflammatory state of PCOS is associated with hyperandrogenemia (103). CRISPR/Cas9 technology has been used to screen genes involved in the regulation of mitochondrial metabolism in GCs, revealing several tumor suppressors with enhanced mitochondrial function, including HBP1, TP53, PTEN, CTNNA2, and WISP1 (104). Among them, HBP1 is involved in regulating mitochondrial production and transcription of the mitochondrial transcription factor (TFAM), a key gene associated with mitochondrial DNA production. Further experiments in mice showed that the knockdown of HBP1 expression enhanced mitochondrial function, reduced apoptosis-related signals in GCs, and promoted follicle development and oocyte maturation (104). Besides, inner mitochondrial membrane peptidase subunit 2 (Immp2l) deficiency leads to mitochondrial dysfunction in GCs, which further leads to increased OS, decreased E2 levels, and altered gene expression levels of Wnt/β-catenin and steroid hormone synthesis pathways, such as CYP19A1, while melatonin is able to reverse these changes (105). The addition of estradiol-17β to the culture medium significantly increased telomere length in GCs, which became more obvious with increasing E2 concentrations (47). Last but not least, as more than 10 enzymes from glycolytic and TCA cycle metabolic pathways were found to be differentially hypo-or hyperacetylated in PCOS, possible therapeutic strategies should be evaluated in the future by targeting miRNAs that specifically regulate the expression of these enzymes (106).

## 8. Conclusion

OS plays an important role in the development of PCOS. This paper reviews and summarizes information on the role of GCs in the pathogenesis of PCOS, the association between OS and POCS, OS markers, antioxidant capacity in patients with PCOS, and the participation of mitochondrial function. To date, most studies have been based on assays performed on serum or FF samples collected from patients with PCOS, and some data have produced conflicting results. More animal and *in vitro* studies are necessary to elucidate the mechanisms by which OS affects fertility in patients with PCOS, and randomized controlled clinical trials are needed to explore more effective targets for the treatment of PCOS.

## Author contributions

LZ contributed to the conception and design of the study and revised the manuscript. YG wrote the first draft of the manuscript. YZ and GW wrote sections of the manuscript. All authors contributed to the article and approved the submitted version.

## Funding

The work was supported by the Natural Science Foundation of Jilin Province, China (YDZJ202201ZYTS084).

## Conflict of interest

The authors declare that the research was conducted in the absence of any commercial or financial relationships that could be construed as a potential conflict of interest.

## Publisher’s note

All claims expressed in this article are solely those of the authors and do not necessarily represent those of their affiliated organizations, or those of the publisher, the editors and the reviewers. Any product that may be evaluated in this article, or claim that may be made by its manufacturer, is not guaranteed or endorsed by the publisher.
